# Sexual Harassment and Coercion in German Academia: A Large-Scale Survey Study

**DOI:** 10.5964/sotrap.9349

**Published:** 2022-12-19

**Authors:** Merle Hoebel, Ana Durglishvili, Johanna Reinold, Daniel Leising

**Affiliations:** 1 Faculty of Psychology, Technische Universität Dresden, Dresden, Germany; Centre for Criminology (Kriminologische Zentralstelle – KrimZ), Wiesbaden, Germany

**Keywords:** sexual harassment, sexual coercion, silence, reporting, academia

## Abstract

We surveyed a large sample (N = 6,217) of students and employees at a German university regarding their experiences as (potential) targets of sexual harassment and/or coercion (SH/C). Participants were asked specific questions depending on whether they had been targets of SH/C themselves, knew someone who had been affected or said they had no such experiences. Pre-registered analyses showed that women were assumed to become targets more often, and actually did become targets much more often (26.7%) than did males (4.7%; odds ratio: 7.45). Men more often had no first- or second-hand knowledge of any SH/C incidents (odds ratio: 1.75). Contrary to what participants assumed they would do if they became targets, only a very small percentage of such experiences were actually reported using the available channels. Most participants who experienced but did not report SH/C said they did not expect that doing so would lead to any consequences. Greater offence severity was associated with a stronger wish to avoid emotional distress by not reporting. Furthermore, reporting often times did not lead to any significant consequences in the majority of cases. Complaint systems against sexual harassment and coercion in academia may be largely dysfunctional. Practical implications are discussed.

## Abstract (plain language summary)

**Background:**

Experiences of sexual harassment and coercion (SH/C) may take many forms, occur in many different contexts, and harm those who become victims tremendously. Academia with its steep hierarchies and strong interpersonal dependencies may constitute a fertile breeding ground for this kind of unethical behaviour.

**Why was this study done?:**

This study was conducted to illuminate the frequencies with which various forms of SH/C (ranging from very mild to very severe) are experienced by students and employees in a German university context. We were also interested in who was most likely to become a victim, and particularly in whether those who became victims would use the available reporting channels, and to what effect.

**What did the researchers do and find?:**

We surveyed more than 4,000 students and employees at a large university in Germany. Participants were asked to describe whether they or someone they knew had ever become the target of some form of sexual harassment or coercion in the context of the university. Approximately 40% affirmed this. Participants assumed that women become targets of such behaviours much more often, which was clearly corroborated by the responses of actual targets. Various types of SH/C offences could be ranked by perceived severity, based on the percentages of participants who said they would file a complaint should they become a target. However, actual reporting fell far short of these expectations: for the most severe forms of SH/C, expected reporting was close to 100%, but fewer than 20% of experiences of this kind were actually reported. Prominent reasons for non-reporting were uncertainty as to whether one’s experiences were severe enough to report, avoidance of further emotional distress, and the expectation that reporting would not lead to any significant consequences. This latter expectation was also largely corroborated by those participants who said they had formally reported their own SH/C experiences.

**What do these findings mean?:**

Our study shows that various forms of sexual harassment and coercion do occur in academia, ranging from relatively mild and common to very severe and rare. Drawing attention to this fact is important because targets are very reluctant to talk about or even report such experiences, which may lead to the misperception (e.g., on the side of institutions) that there is no problem to be addressed. According to our study, complaint systems may be largely dysfunctional because (a) victims have little trust in them and (b) they too often prove ineffective when used. We discuss potential remedies like awareness training. Overall, strengthening prevention efforts may be the most promising route to alleviating the problem of SH/C in academia.

## Highlights

- Women were assumed to—and did—become targets of sexual harassment and coercion (SH/C) much more often than men (odds ratio: 7.45).
- Milder forms of sexual harassment were common.
- Even the most severe experiences of sexual harassment or coercion were rarely reported, and in many cases reporting did not lead to consequences.
- The wish to avoid further emotional distress tends to impede reporting of the most severe SH/C experiences.

Extensive research has established that sexual harassment is common and can happen everywhere (Bucchianeri et al., 2013; Heise et al., 2002; Müller et al., 2004; Turchik et al., 2016). Sexual harassment and coercion (hereafter abbreviated as SH/C) take place across all social strata, within different types of organisations, and in a variety of relationships between victim and offender. In recent years, several severe cases of sexual misconduct at universities have become public (e.g., P-magazine, 25. October 2022). In the present study, we take a detailed look at the respective situation at a large university in Germany, focusing specifically on the reporting of sexual harassment and coercion, as well as the consequences of such reporting.

Academic environments in general may be particularly conducive to all kinds of unethical behaviours (including SH/C; Ilies et al., 2003) because they tend to entail steep formal hierarchies (e.g., between professors and students) and strong interpersonal dependencies (e.g., between senior and junior researchers; Täuber et al., 2022). Hierarchies at academic institutions in Germany are perceived to be even steeper than in other countries (Kreckel, 2016; Ohm, 2023), while also having only weak mechanisms in place to professionally assess, prevent, and possibly sanction occurrences of SH/C. Furthermore, academia is characterised by an unequal distribution of power between men and women (Goastellec & Pekari, 2013). This is relevant because research unequivocally demonstrates that the vast majority (80 – 99%) of offenders are male and the vast majority of victims are female (Dartnall & Jewkes, 2013; Feltes et al., 2012; Mense et al., 2022).

Many studies have investigated sexual harassment and coercion at American universities (Cantor et al., 2019). The number of such studies about the German academic sector is comparably small and the existing ones (e.g., Carl von Ossietzky University Oldenburg, 2012; University of Greifswald, 2018) used relatively small samples. Nationwide surveys concerning the topic (such as the study by Kearl, 2018 for the US) do not exist, according to our knowledge and the last such study in Germany using a big sample was Feltes et al.’s “Gendercrime” study in 2012 (according to self-disclosure in the text, their sample was not representative). So, while we do have some knowledge about the prevalence of SH/C at German universities, this knowledge is fairly limited. With the present study, we aim to broaden this knowledge base considerably, by using an unusually large sample that includes both students and employees. Further we assess some key aspects of the problem, such as the (mal-) functioning of complaint systems.

## Definitions

In the relevant directive by the European Union (EU), sexual harassment is defined as “any form of unwanted verbal, non-verbal or physical conduct of a sexual nature [...] with the purpose or effect of violating the dignity of a person, in particular when creating an intimidating, hostile, degrading, humiliating or offensive environment" (EU Directive 2006/54/EC). Corresponding national laws are in force. In the present study, we also ask participants if they ever received any unsolicited pornographic materials. For the sender such behaviour is defined as punishable under the German Criminal Code section 184 (“Verbreitung pornographischer Inhalte”). Furthermore, the *sexual coercion* (“Sexuelle Nötigung”) of a person, which includes the criminal offence of *rape* (“Vergewaltigung”), is defined in section 177 of the German Criminal Code as a sexual act or forcing of such an act against the noticeable will of the target. Coercion is also present if the target is not able to form or express consent or if they are being threatened with significant negative consequences should they refuse. In the present study, we take a rather comprehensive approach by assessing the entire range of harassment and coercion offences, ranging from rather mild ones (like offensive jokes) to very severe ones like rape. When referring to this entire range comprehensively, we use the term “sexual harassment/coercion” (SH/C).

## Prevalence and Risk Factors

In this section, we will briefly summarise the existing research literature on the prevalence of SH/C, and on factors that increase the risk of becoming a victim. Unsurprisingly, most or even all of these factors (e.g., gender, age, institutional role) are correlated with the power that the potential victim has relative to the potential offender: the less power potential victims have the more likely it is that they will be harassed (Ilies et al., 2003). Please note that the research literature on these topics is not very consistent in terms of definitions and methodology. For example, it has been found that the reported prevalence of SH/C depends quite strongly on the way in which survey questions are phrased: generally asking for experiences of SH/C leads to lower prevalence, while asking for specific forms of SH/C (e.g., Have you experienced catcalling) leads to higher prevalence (Ilies et al., 2003). Despite these variations, some fairly consistent conclusions may be drawn from the available research.

The most extensively studied risk factor, by far, is gender. Overall, women have a higher risk of becoming victims of SH/C than men. List and Feltes (2015) found that female students at a German university were almost three times as likely (13%) as men (4.6%) to report experiencing some sort of SH/C. Likewise, in a US-study involving students from nine different universities, women were significantly more likely to report having experienced sexual coercion (female: 7.7%, male: 5.8%) or sexual harassment (female: 28%, male: 13.2%) (Krebs et al., 2016). Probably as a result of the consistently found gender imbalance in prevalence, the majority of studies on SH/C focus on women as victims. In the Germany-wide “Gendercrime” study, 54% of female students stated that they had already experienced some form of sexual harassment, and 3.3% stated that they had been victims of sexual coercion (Feltes et al., 2012). A similar result was obtained by researchers in the USA who, in a meta-analysis of 71 studies, investigated the sexual harassment experiences of women in academia and found a prevalence of 58% (Ilies et al., 2003). However, in the study by List and Feltes (2015) only 19.2% of women reported that they had experienced sexual harassment in their academic, school or professional environment. Focusing on the most severe forms of SH/C, different studies found that 0.6% to 5.5% of female students said they had been a victim of rape while 1.1% to 4.3% reported an attempted rape (Feltes et al., 2012; Kury et al., 2004; Müller et al., 2004).

Another relevant factor besides gender is a potential victim’s age, because previous research suggests that young adults are most likely to become targets of SH/C (List & Feltes, 2015). Müller et al. (2004) found that women between the ages of 18 and 24 were more than three times as likely to be targeted than women between the ages of 45 and 54, and more than twice as likely than women between 35 and 44. In a Europe-wide study, Latcheva (2017) identified younger age (18-28 years) as the strongest among ten socio-demographic predictors of SH/C. Two factors come to mind when trying to explain this pattern of findings. First, younger people are considered to be more attractive by people of all ages (Korthase & Trenholme, 1982; Wernick & Manaster, 1984), and the youth of female targets is an especially strong predictor of sexual interest in heterosexual men (Kenrick & Keefe, 1992). The latter finding is consistent with an evolutionary perspective of human psychology and behaviour (Buss, 1989). Second, younger age is reliably associated with lower power in institutions, which means that younger persons are exposed to a greater number of others who might abuse the relatively greater power that they have.

Within the academic context, being a *student* is associated with having little institutional power compared to most other actors (e.g., professors with grading authority). Thus, being a student may be associated with a relatively high risk of being victimised (Edwards & Greenberg, 2011; MacKinnon, 1979; Naezer et al., 2019). However, power differentials among employees in academia (e.g., between professors and PhD students) are steep as well, and members of the same work group often spend a lot of time together. Both factors may bring about a risk of being sexually harassed for lower-ranking employees in particular (De Coster et al., 1999). In a population-representative study by Müller et al. (2004), female university students reported being the target of SH/C relatively more often (13%) than female university employees did (5,1%) (List & Feltes, 2015). An effect with the same direction was found in a study at a German university in which 40% of students and 30% of employees stated that they had been the target of some type of sexual harassment (University of Greifswald, 2018). Another study from the US also found lower victimisation rates of employees compared to students (Bondestam & Lundqvist, 2020). In the present study, we investigate prevalence and predictors of SH/C experiences for students and employees at the same university.

Apart from the factors described here, various authors point out that being part of a sexual or ethnic minority further increases the likelihood of ones’ victimization (Kammer-Kerwick et al., 2021; List & Feltes, 2015; Naezer et al., 2019). These points were not included in this study due to data privacy concerns but should be further explored in the future.

## Voice and Silence

The present study also investigates the use and the effectiveness of reporting channels to counteract SH/C in academia. This issue is important for several reasons: First, actual reporting rates will directly influence the extent to which relevant agents in the system (e.g., university leadership) recognize SH/C as a problem. Second, reporting is a necessary condition for SH/C to be sanctioned. We investigate how often SH/C experiences are reported, to whom, and with what effect, and what the reasons are for *not* reporting. These issues directly concern the concepts of *voice* and *silence* in organisations. Notably, silence is not the same as the absence of voice since people might abstain from raising their voice because they simply have nothing to report (Knoll et al., 2016). Regarding sexual harassment in the workplace, Bergman and colleagues define voice as “the act of telling an organizational authority […] about unwanted or offensive sex-related behaviour” (Bergman et al., 2002, p. 231). In the present study, however, we use a somewhat broader concept of voice, which also includes any attempts to draw the attention of agents *outside* of an organisation (e.g., legal authorities) to problems that exist within the organisation. This is important because many occurrences of SH/C constitute criminal offences. Regarding the reporting of such offences, formal reporting (e.g., to the police or a supervisor) may be distinguished from informal reporting (e.g., talking to a friend or colleague) (Mennicke et al., 2022). Both types of reporting are assessed in the present study. While sexual harassment is common, reporting it is rare (European Union Agency for Fundamental Rights [FRA], 2014; Feltes et al., 2012). According to one study, nine out of ten victims of sexual harassment never formally report their experiences (Hershcovis et al., 2021). In an EU-wide study only 4% of victims of sexual harassment approached the police or a supervisor at work with a complaint (FRA, 2014). Other studies on sexual harassment found somewhat higher rates of formal reporting (5-30%; McDonald, 2012). Surely, these relatively low numbers may partly be explained in terms of the perceived mildness of many instances of sexual harassment. A study looking at informal ways of reporting (e.g., to friends or colleagues) found that about 30% of female university students who had become targets of sexual harassment said they had *not* shared their experiences with anyone, but more than 45% said so when they had become targets of sexual coercion (Feltes et al., 2012). This suggests that greater offence severity may have some kind of *inhibitory* effect on reporting. In the present study, we address the link between offence severity and reporting rates, as well.

However, a plethora of other factors may also explain why victims of SH/C abstain from sharing their respective experiences with others: many people who become targets of sexual harassment are not sure if their experiences are severe enough to be reported (Cantor et al., 2015; Krebs et al., 2007). Also, the perceived ineffectiveness of available reporting systems keeps victims from using them (McDonald, 2012). One of the factors contributing to this may be the fact that sexual offences are difficult to prove (e.g., in court) because they often take place with no-one present but the victim and the perpetrator. Victims thus rightfully fear being sued for defamation. In fact, research has shown that filing complaints often *does* have significant negative consequences for those filing them since retaliation attempts and problematic behaviours (e.g., bullying) may even increase rather than decrease, as a consequence of reporting them (Bergman et al., 2002; Cortina & Magley, 2003; Kiewitz et al., 2016; McDonald, 2012). Apart from the actual risk of being retaliated against by the accused, victims may also fear that perpetrators who are powerful and visible members of the organisation are likely to be protected against accusations by the same (McDonald, 2012).

Other important factors that may keep victims from reporting are *feeling ashamed* for what happened to them (Feltes et al., 2012), an organizational *climate* in which speaking up appears to be unwanted (Morrison et al., 2011) or a simple *lack of knowledge* regarding available reporting channels (FRA, 2014).

## The Present Study

The present study aims to broadly examine various aspects of SH/C in academia. We assessed participants’ estimates of how frequent such violations are, and who is particularly prone to becoming a target. Then participants were asked more specific questions depending on whether they had been targets of SH/C themselves (“Path A”), knew someone who had been affected (“Path B”) or stated they had no personal knowledge of any such experiences (“Path C”). For Paths A and B, we assessed the actual prevalence of various types of SH/C and further investigated whether these experiences had been reported and, if yes, to what effect. If experiences were not reported, we asked participants why this did not happen.

The overall sample was randomly divided into a smaller “exploration sample” and a larger “hold-out sample”. The first of these was used to freely explore the data for any noteworthy effects. Based on this first set of analyses with the exploration sample, we developed a more specified plan for analyses to be conducted with the hold-out sample, in a more confirmatory fashion. This plan was pre-registered (https://osf.io/3uma5) and included the following four hypothesis tests:

**Hypothesis 1.** Expected prevalence: Women are assumed to become targets of sexual harassment or coercion more often than are men.

**Hypothesis 2.** Expected prevalence: Participants later choosing Path A (“I have been a target myself”) provide higher estimates than participants who later choose Path B (“I have not been a target myself but personally know someone who has”), which in turn provide higher estimates than participants who later choose Path C (“I do not know anyone who has been a target”). In other words, one’s prevalence estimate increases the more immediate one’s experience with the issue is.

**Hypothesis 3.** Actual prevalence: Women choose Path A (as opposed to B or C) more often than do men. In other words, women say that they themselves have become targets of sexual harassment and/or coercion more often than do men.

**Hypothesis 4.** Actual prevalence: Men choose Path C (as opposed to A or B) more often than do women. In other words, men say more often that they do not have any first- or second-hand knowledge of any sexual harassment and/or coercion incidents.

In addition to these tests, we also specified and pre-registered a number of broader, more descriptive analyses, which we consider to be at least equally important (e.g., regarding reasons for not reporting SH/C experiences). With these analyses, it is more the *overall pattern of responses* (e.g., to different variables) that is of interest, which is why we abstain from additional statistical testing.

## Method

### Procedures

#### Questionnaire Design

In order to make the target concept as unambiguous as possible for all participants in the study, we presented them with the following definition up front: In the context of this survey, we use the term “sexual boundary violations” (in this article referred to as SH/C) to refer to the following behaviours:

- Suggestive remarks or jokes with sexual/obscene content
- Intimidating stares
- Unwanted requests for sexual acts
- Coercing or "pressuring" someone to engage in sexual activity
- Offering favours/benefits in exchange for sexual activity
- Distributing intimate material in which the targeted person is identifiable
- Receiving unsolicited pornographic material
- Unwanted physical approach and/or touching (e.g., fondling, hugging), even if the touching seems to be accidental
- Use of physical force to assert sexual interests (up to and including rape)

This definition is derived from section 3(4) of the *German General Act on Equal Treatment* but is phrased in more understandable terms and additionally includes aspects of sexual assault and rape in order to cover the whole range of SH/C severity.

Following this definition, participants were first asked to separately estimate the prevalence of SH/C for men and women studying and/or working at the university. We also asked them whether they thought that moving to online learning/work during the COVID-19 pandemic had affected the prevalence. This was followed by a question asking whether participants thought they had been the target of SH/C themselves (Path A), had not been a target but personally knew someone who had (Path B), or had no such knowledge (Path C). Based on their responses participants were assigned to one of three mutually exclusive sets of questions (the “paths” just mentioned) pertaining to their respective experiences. Participants in Paths A and B were asked to describe the relevant event that they recalled most clearly, and to provide more details on it (e.g., the specific type of SH/C, the target’s relationship with the offender, whether the event was reported and – if yes – to what effect). Participants in Path C were asked to predict how they would behave if they ever became a target of any kind of SH/C, especially whether they would formally report these experiences or recommend doing so (if someone else became a victim).

This was followed by a final set of questions, including sociodemographics, to be answered by all participants. The questionnaire was available in English (*n* = 342 before exclusion) or German (*n* = 5,875 before exclusion). Even though the survey took less than ten minutes to complete, the dataset was still so complex that not all variables could be used for the present analyses. In this paper, we focus on the research questions that we found to be of the highest importance. The questionnaire was designed specifically for this study and is available as an online supplement.

#### Recruitment

All students and employees of the university were contacted via their official university email addresses. The email briefly explained the topic of the survey, made clear that everyone could participate (i.e., irrespective of whether one had ever been a target of SH/C), and assured the confidential treatment of all data, and anonymity. A total of 29,797 emails were sent successfully.

#### Samples

We obtained responses from 6,217 participants (see Table 1). On November 30^th^, 2021, we drew a random 30% (*n* = 1,837) subsample from these cases, to be used in a first, exploratory round of analyses. Based on these analyses, specific hypotheses and an analysis plan were developed and pre-registered (available as an online supplement). The remaining 4,380 cases (the “hold-out sample”) were retained for confirmatory analyses to be conducted later on. The results section of the present paper reports the outcome of analyses conducted with this latter sample. Unfortunately, participants self-identifying with a gender apart from male or female could not be included in most of the analyses because of the very small sample size.

**Table 1:** Sample Characteristics

Variable / Specification	Exploration sample	Hold-out sample
**Cases analysed**	1,820	4,333
Gender		
Male	508 (27.9%)	1,139 (26.3%)
Female	728 (40.0%)	1,695 (39.1%)
Diverse/Other	13 (0.7%)	43 (1.0%)
Missing	571 (31.4%)	1,456 (33.6%)
Role		
Student	660 (36.3%)	1,586 (36.6%)
Employee	410 (22.5%)	917 (21.2%)
Both	179 (9.8%)	379 (8.7%)
Missing	571 (31.4%)	1,451 (33.5%)
Age		
Mean (Mode)	27.53 (23)	27.90 (24)
Min; Max	18; 64	18; 65
Quartiles (25, 50, 75)	22.0; 25.0; 30.0	22.0; 25.0; 31.0
*SD*	8.33	8.72
Missing	573 (31.5%)	1,456 (33.6%)
Decline to answer	116 (6.4%)	238 (5.5%)

The university from which the sample was drawn is a technical university with approx. 32,000 students and 9,000 employees (as of 2019; Pressestelle Technische Universität Dresden, 2022). The university is one of the 30 largest universities in Germany. About one third of all students who pursue an academic education in Germany are enrolled at one of these 30 universities (Hochschulkompass, 2022). It offers a wide range of 124 degree programs from various fields such as natural sciences, engineering, humanities and medicine.

#### Data

Due to the sensitive nature of the data and the explicit assurances of anonymity and confidentiality that were given to the participants, the raw data is not made publicly available. However, the complete data will be stored in a persistent fashion at a secure server located at TU Dresden.

#### Exclusion Criteria

Exclusion criteria were specified and applied before any of the analyses presented here took place. For legal reasons, participants stating that they were younger than 18 years were excluded from analyses. Participants reporting ages older than 70 were also excluded as outliers in terms of age. Furthermore, we excluded participants who reported that their own SH/C experiences took place or began before they were 18, because in these cases it was not clear enough whether the respective events had taken place within the academic context. Beyond that, ambiguous age information (e.g., ranges) was replaced with missing values but the remaining information on such cases was retained for analyses. After applying these criteria 47 participants were excluded, accordingly *n* = 4,333 cases from the hold-out sample were analysed. No other exclusion criteria were applied.

## Results

### Estimated Prevalence

In the first part of our survey, we asked all participants to estimate the percentage of people at the university who had experienced some form of SH/C, and the percentage of those who had formally reported their respective experiences.

In accordance with our pre-registration, we conducted a repeated measures ANOVA with the gender of potential victims as a within-subject factor and participants’ age, gender, institutional role (student, employee, or both) and “path” chosen later in the questionnaire (A: been target myself, B: know someone who has been a target, C: do not know anyone who has been a target) as between-subject factors. The estimated overall prevalence of SH/C was predicted from these variables. As we had expected based on our analyses of the exploratory sample, there was a significant effect of target gender, *F*(1, 2029) = 228.82, *p* < .001, $\eta_{p}^{2}$ = .101, and a significant effect of path, *F*(2, 2029) = 107.68, *p* < .001, $\eta_{p}^{2}$ = .094. Participants did expect women to become targets more often, and they assumed higher prevalence the more immediate their own experience with being targeted was. Both effects were in line with our pre-registered hypothesis 1. In addition, we also found a significant interaction between target gender and path, *F*(2, 2029) = 56.154, *p* < .001, $\eta_{p}^{2}$ = .052. The same effect had already occurred in the exploratory sample, but we did not make it part of our hypotheses because we found it somewhat difficult to interpret. The other predictors did not have any relevant influence of their own. Descriptively, it was noteworthy that expected reporting rates were very low (below 20%).

### Actual Prevalence

In the hold-out sample, 20.2% (*n* = 711) of the participants who responded to our key question about their own respective experiences said that they had been the target of some kind of SH/C in the past (Path A). Another 22.5% (*n* = 793) said they personally knew someone who had been a target (Path B). So, approximately 40% of the sample reported having either first-hand or second-hand knowledge of some form of SH/C. Note that these categories were mutually exclusive, in order to minimise the time it would take participants to complete the questionnaire: Participants choosing the first response option (Path A: being the target) might also have had knowledge of incidents in which others became targets, but we did not ask these participants about their knowledge in this regard. Thus, it is likely that the number of participants who knew *others* that had been targets is underestimated somewhat by our study. Another 57.3% (*n* = 2,016) of participants in the hold-out sample reported that they had no knowledge of any sexual harassment or coercion incidents.^1^1Note that we had explicitly asked the participants about their experiences within the university context, but free format responses made it clear that some (few) participants had nevertheless described experiences they had had before they took up their studies. Given that the number of these cases was very small (< 30 in each path), we decided to ignore the issue and treat this data as random error.

Note that the actual overall prevalence we found in Path A was considerably smaller than the estimated ones (for male targets: estimated = 16.7%, actual = 4.7%; for female targets: estimated = 47.8%, actual = 26.7%; see Tables 2 and 3). Due to differing response formats (count vs metric), a statistical comparison of these rates was not possible. However, we discuss possible reasons for this discrepancy below.

**Table 2:** Estimated Prevalence of Sexual Harassment and Coercion Separated by Person Estimating and Target Gender

Gender of person estimating	Estimated percentage of affectedness		∅ per row	Estimated percentage of formal reporting when affected		∅ per row
	Among Males	Among Females		Among Males	Among Females	
Male	15.7 (17.0) *n* = 900	43.9 (29.3) *n* = 930	29.8	7.1 (12.9) *n* = 847	16.1 (16.1) *n* = 890	11.6
Female	16.4 (15.0) *n* = 1,311	48.8 (28.8) *n* = 1,379	32.6	6.6 (12.2) *n* = 1248	14.0 (14.1) *n* = 1,333	10.3
Other (not specified or diverse)	19.3 (19.3) *n* = 535	51.6 (30.3) *n* = 565	35.5	6.9 (13.2) *n* = 506	17.3 (19.3) *n* = 528	12.1
∅ per column	16.7	47.8		6.8	15.3	

*Note.* Mean estimated percentages, with *SD* in parentheses. Numbers in the bottom row are weighted for *n*. ∅ are weighted.

**Table 3:** Prevalence Separated by Gender

Response option	Male	Female	Other/ diverse	Total (reporting gender)	Total (all)
Yes, I was the target of such behaviours myself	53 (4.7%)	452 (26.7%)	7 (16.3%)	512 (17.8%)	711 (16.4%)
Yes, I know one or several persons who were targets of such behaviours	306 (26.9%)	304 (17.9%)	14 (32.6%)	624 (21.7%)	793 (18.3%)
No, I do not know such a case	780 (68.5%)	939 (55.4%)	22 (51.2%)	1741 (60.5%)	2,016 (46.5%)
Total	1,139 (39.6%)	1695 (58.9%)	43 (1.5%)	2,877 (100%)	4,333 (100%)

### Age at the Time of Victimisation

Participants in Paths A and B were asked how old the affected person was when the SH/C took place. Only a relatively small percentage answered this question: in Path A (*n* = 410), the mean reported age was *M* = 24.2 (*SD* = 6.1, Med = 22, Min = 18, Max = 52; 90% percentile = 32.9). In Path B (*n* = 341), the mean reported age was *M* = 23.54 (*SD* = 4.7, Med = 22, Min = 18, Max = 50, 90% percentile = 29.0). Reports in both paths were highly similar, despite these being two independent samples of participants. This suggests a high level of validity: The vast majority of SH/C experiences occur at a time when victims are in their twenties.

### Prevalence Separated by Gender

Table 3 shows the reported prevalence of SH/C separated by gender. Note that the inclusion of the gender variable in this analysis reduced the number of usable cases (to *n* = 2,834) because 1,499 participants had not reported their gender. Even at first glance, a striking gender difference becomes evident: More than a quarter of women (26.7%) reported to have been sexually harassed and/or coerced, while this was the case for 4.7% of men only. Female participants (452) compared to male participants (53) were 8.53 times more likely to have been victims of the SH/C, even though the female-to-male ratio among the remaining participants was only (1,243/1,086=) 1.14.

Thus, being female was an extremely strong and statistically significant predictor of reporting to have been a target, odds ratio = 8.53/1.14 = 7.45, 99% CI [5.05, 10.99]. Note further that the number of men who reported having no personal knowledge of any SH/C incidents (Path C; *n* = 780) by far exceed the number of men who reported having such knowledge (Path A+B; *n* = 359), whereas this ratio was more balanced among women (939/756). The respective odds ratio was (2.17/1.24 =) 1.75, 99% CI [1.42, 2.15]. We consider this finding important because it suggests that men may not have much information regarding the problem of SH/C.

### Prevalence Separated by Type of SH/C

Table 4 displays the frequencies with which the various forms of SH/C were experienced in the two samples, both by the persons who said they had been targets themselves (Path A), and by the persons who said they had heard about others’ experiences (Path B). There was a great variance in how often the individual categories were endorsed: “Suggestive remarks” and “intimidating stares” were reported to be very frequent forms of harassment, whereas other forms such as “use of physical force” were much rarer. We did not obtain explicit ratings of incident severity, but it was nevertheless obvious that more severe forms of harassment were reported as being among the least common. We will return to this issue further below (see “Overall Severity”).

**Table 4:** Prevalence Sorted by Type of Sexual Harassment/ Coercion (SH/C)

Type of SH/C	Path A	Path B
Suggestive remarks or jokes with sexual/ obscene content [SH]	481 (80.8/11.1%)	544 (78.3%)
Intimidating stares and/or "checking out"/examining/ gazing [SH]	381 (64.0/8.8%)	358 (51.5%)
Unwanted physical contact and/or touching (e.g., fondling, hugging), even if the touching seems accidental [SH]	272 (45.7/3.2%)	228 (32.8%)
Unwanted suggesting of sexual acts [SH]	97 (16.3/2.2%)	81 (11.7%)
Receiving unsolicited pornographic material [SH]	67 (11.3/1.6%)	76 (10.9%)
Offering favours/benefits in exchange for sex [SH]	62 (10.4/1.4%)	59 (8.5%)
Forcing or "pressuring" someone to engage in sexual activity [SC]	52 (8.7/1.2%)	51 (7.3%)
Use of physical force to assert sexual interests (up to and including rape) [SC]	35 (5.9/0.8%)	36 (5.2%)
Distributing intimate material in which target is identifiable [SH]	19 (3.2/0.4%)	26 (3.7%)
Relative to (absolute numbers)	595/4,333	695

*Note*. The first percentage in parentheses is relative to all participants in the respective path (A: been target / B: know a case). The second percentage is relative to all participants regardless of path. Endorsing multiple responses was possible.

In terms of absolute numbers of reported occurrences in Path A, it should be noted that 35 participants (23 female, 3 male, among 26 who reported their gender) indicated that someone had subjected them to physical force to assert his or her own sexual interests, 62 (51 female and 5 male, among 56 who reported their gender) indicated that someone had offered them favours/benefits in exchange for sexual activities, and 52 (38 female, 4 male, among 42 who reported their gender) said they had been pressured to engage in sexual activity. These results indicate that some relatively severe kinds of SH/C *have* happened to a sizable number of participants. Note that these categories are not mutually exclusive, so smaller numbers may have been (partly) included in larger ones (e.g., due to simultaneous occurrence of different types of SH/C in the same incident).

In the present study, seven response options can be summarized under the umbrella term sexual harassment, including, for example, "suggestive remarks or jokes..." and "unwanted physical touch..." (see Table 4 for a complete overview). In Path A, at least one of these categories was selected by 13.6 percent of all respondents, 26.4 percent of all female respondents (*n* = 1,695 in total), and 4.6 percent of all male respondents (*n* = 1,139 in total), indicating that they had experienced some type of sexual harassment in the university context. The two categories "forcing or pressuring someone to engage in sexual activity" and "use of physical force to assert sexual interest..." can be summarized as sexual coercion and were selected by 1.4 percent of all respondents, 2.5 percent of all female respondents (of *n* = 1695 in total) and 0.4 percent of all male respondents (of *n* = 1,139 in total) in Path A. A version of this table separated by gender may be accessed as an online supplement.

### Reporting

Table 5 displays the numbers and percentages of experiences that were formally reported in some way, separately for the various types of SH/C experienced by the respective target. Again, a version of this table separated by gender may be accessed as an online supplement.

**Table 5:** Number of Formal Reporting Sorted by Type of Sexual Harassment/ Coercion (SH/C)

Type of SH/C	Path A		Path B	
	Cases	Formal report	Cases	Formal report
Suggestive remarks or jokes with sexual/ obscene content	481	10 (2.1%)	544	26 (4.8%)
Intimidating stares and/or "checking out"/examining/ gazing	381	8 (2.1%)	358	14 (3.9%)
Unwanted physical contact and/or touching (e.g., fondling, hugging), even if the touching seems accidental	272	8 (2.6%)	228	15 (6.6%)
Unwanted suggesting of sexual acts	97	8 (8.3%)	81	11 (13.6%)
Receiving unsolicited pornographic material	67	5 (7.5%)	76	4 (5.3%)
Offering favours/benefits in exchange for sex	62	4 (6.5%)	59	4 (6.8%)
Forcing or "pressuring" someone to engage in sexual activity	52	6 (11.5%)	51	8 (15.7%)
Use of physical force to assert sexual interests (up to and including rape)	35	5 (14.3%)	36	4 (11.1%)
Distributing intimate material in which target is identifiable	19	2 (10.5%)	26	2 (7.8%)

Note, however, that there is no direct correspondence between types of offences and reporting here because the former was assessed using a multiple-choice format: for example, there were *n* = 58 participants who said that someone had offered them favours in exchange for sex, and of these *n* = 4 said that they had formally reported some of their experiences. This does not mean that the *reported* experiences were of that particular type, because we had not explicitly asked the participants about the exact *content* of their reports. It only means that the person doing the reporting had also experienced this specific form of harassment (possibly amongst other forms), according to the person completing the survey.

Overall reporting percentages were very low. Even participants who said they had experienced the most severe forms of sexual harassment did not file any formal complaints in more than 85% of cases. For example, of the 35 participants who said that they had been the target of “physical force to assert sexual interests”, only five (14.3%) stated that they had formally reported this experience. We will address reasons for non-reporting further below.

### Comparison With Assumed Reporting Rates

Given that our study put particular emphasis on the effectiveness of reporting channels, it seemed important to compare the actual reporting rates found for participants who *did* have first- or second-hand experience with being sexually harassed (Paths A and B) with the reporting rates predicted by participants *without* such experiences (Path C). Specifically, we asked the latter to state whether they *would* file an official internal (e.g., reporting office of the university) or external (e.g., police) report if they ever became victims of SH/C, and whether they would recommend doing so to someone else who became a target. Results are displayed in Table 6.

**Table 6:** Assumed Reporting Behaviour by Participants With no Personal Knowledge of Actual Sexual Harassment/ Coercion (SH/C; Path C)

Type of SH/C	Expected reporting (%)							
	No reporting		Yes, report internally		Yes, report externally		Reporting total (int. + ext.)	
	Self	Other	Self	Other	Self	Other	Self	Other
Suggestive remarks or jokes with sexual/ obscene content	82.5	53.6	16.8	45.3	0.7	1.1	17.0	46.4
Intimidating stares and/or "checking out"/examining/ gazing	87.5	55.3	11.9	43.3	0.6	1.5	12.5	44.7
Unwanted physical contact and/or touching…	54.8	23.0	37.1	61.9	8.1	15.0	45.2	77.0
Unwanted suggesting of sexual acts	22.4	6.6	58.2	62.2	19.4	31.2	77.6	93.4
Receiving unsolicited pornographic material	28.9	*	40.0	*	31.1	*	71.1	*
Offering favours/benefits in exchange for sex	16.7	5.5	68.4	70.4	14.9	24.1	83.3	94.5
Forcing or "pressuring" someone to engage in sexual activity	5.1	1.4	42.4	34.6	52.5	64.0	94.9	98.6
Use of physical force to assert sexual interests (incl. rape)	1.3	0.5	5.3	6.6	93.4	92.9	98.7	99.5
Distributing intimate material in which target is identifiable	4.4	*	14.8	*	80.8	*	95.6	*

*Note*. Sample sizes to which the percentages refer differ slightly as this was not a compulsory item. *no data available, due to a glitch in survey construction. The answers were mutually exclusive within each type of SH/C.

There were large discrepancies between predicted and actual reporting rates. Particularly instructive in this regard: For the most severe transgressions, participants in Path C assumed reporting rates of beyond 80 and often close to 100 percent. However, the actual reporting rates for these experiences never reached 20 percent (see Table 5). This suggests that those unaffected by SH/C so far seem to have a hard time imagining what actually becoming a victim feels like, including the possible emergence of emotional barriers (e.g. shame, guilt) against reporting such experiences.

### Reasons for not Formally Reporting Experiences With SH/C

Given the vast discrepancies we found between predicted and actual reporting rates, one needs to ask: *why* did the majority of participants in Paths A and B decide to abstain from formally reporting their experiences, even those of the most severe kind? Table 7 displays the outcome of the respective analyses (for Path A). Overall (column “total”), these participants’ most prominent reason for not reporting was uncertainty as to whether the incidents in question were severe enough to warrant reporting (79.1%). This aligns with the fact that most of the experiences described by our participants in the survey seemed to be comparatively mild. The second most selected reason for not reporting was the expectation that doing so would not lead to any actual consequences (63.3%). Other important reasons were uncertainty as to who SH/C may be reported to (40.7%), to protect oneself from negative consequences (24.6%), and to avoid causing trouble to the offender (17.6%). The respective percentages for Path A correlated almost perfectly (*r* = .98) with those for Path B (not displayed here), which we interpret as an indication of their validity, especially given that they were provided by two independent samples of hundreds of participants. The remaining columns of Table 7 reflect the influence of the various reasons for non-reporting separated by the overall severity of the respective transgressions. These are addressed in the next section.

**Table 7:** Reasons for not Formally Reporting Sexual Harassment/ Coercion (Path A: Participants Who Had Been Targets Themselves)

Reason	Total	Separated by severity							
		12.5	17.5	45.2	71.1	77.6	83.3	94.9	98.7
Because it was unclear whether the case was serious enough to report	430 (80.2%)	18 (72.0%)	180 (82.2%)	122 (80.3%)	16 (84.2%)	31 (79.5%)	28 (75.7%)	16 (69.6%)	16 (61.5%)
There would probably not have been any consequences anyway, so reporting it would have been pointless	348 (64.9%)	9 (36.0%)	138 (63.0%)	88 (57.9%)	12 (63.2%)	28 (71.8%)	28 (75.7%)	17 (73.9%)	22 (84.6%)
Because it was unclear who could have been approached	222 (41.4%)	6 (24.0%)	80 (36.5%)	63 (41.4%)	7 (36.8%)	23 (59.0%)	23 (62.2%)	10 (43.5%)	8 (30.8%)
Fear of negative consequences	136 (25.4%)	3 (12.0%)	44 (20.1%)	42 (27.6%)	5 (26.3%)	9 (23.1%)	15 (40.5%)	5 (21.7%)	10 (38.5%)
In order not to cause trouble for anyone (i.e., the perpetrator)	96 (17.9%)	2 (8.0%)	36 (16.4%)	28 (18.4%)	5 (26.3%)	6 (15.4%)	12 (32.4%)	2 (8.7%)	4 (15.4%)
Because of the perception that in the university culture, SH/C are not wanted to be discussed	50 (9.3%)	0 (0.0%)	22 (10.0%)	15 (9.9%)	0 (0.0%)	0 (0.0%)	5 (13.5%)	3 (13.0%)	4 (15.4%)
The incident was so emotional, unpleasant or painful that I did not want to relive it	52 (9.7%)	0 (0.0%)	11 (5.0%)	11 (7.2%)	1 (5.3%)	2 (5.1%)	5 (13.5%)	8 (34.8%)	12 (46.2%)
To be able to use the knowledge against the offender at a later time	4 (0.7%)	0 (0.0%)	2 (0.9%)	1 (0.7%)	0 (0.0%)	0 (0.0%)	1 (2.7%)	0 (0.0%)	0 (0.0%)
Of (participants that answered)	536	25	219	152	19	39	37	23	26

*Note.* Severity is the percentage of participants in Path C who said they would formally report this type of behaviour if they became the target of it (see “Overall severity”). We assigned each participant the respective score for the most severe transgression they said they had experienced: Intimidating stares (12.5), suggestive remarks (17.5), unwanted physical contact (45.2), receiving pornographic material (71.1), suggesting sexual acts (77.6), offering favours in exchange for sex (83.3), exerting pressure (94.9), use of force (98.7). Distribution of intimate material (95.6) was omitted here, due a very small sample size *n* = 8 on which the estimates were based. The answers were not mutually exclusive.

### Overall Severity

We investigated the existence of an implicit severity continuum on which the various SH/C offences could be ranked. Table 8 displays the correlations between the overall frequencies of actual SH/C experiences (row 1: Path A, row 3: Path B), the percentages of such experiences that were formally reported (row 2: Path A, row 4: Path B), and the percentages of such experiences that participants in Path C expected to report should they (row 5) or someone they know (row 6) endure them. These analyses re-use some of the data reported in Tables 4, 5 and 6 above. The “cases” on which these correlations are based are the nine (seven^2^2Due to a glitch in survey construction, there was no data collected for the items “Receiving unsolicited pornographic material” and “Distributing intimate material [...]”.) different types of SH/C, with the values obtained for each SH/C type and variable reflecting hundreds of observations.

**Table 8:** Correlations Between (Actual and Expected) Frequencies and Reporting Rates of Different Types of Sexual Harassment/ Coercion (SH/C) Experiences

Index	Path	Target	Variable	(2)	(3)	(4)	(5)	(6)
(1)	A	Self	Actual frequency	-.85	.99	-.58	-.97	-.97
(2)	A	Self	Actual reporting (%)		-.81	.72	.91	.83
(3)	B	Other	Actual frequency			-.58	–.93	-.95
(4)	B	Other	Actual reporting (%)				.68	.79
(5)	C	Self	Expected reporting (%)					.98
(6)	C	Other	Expected reporting (%)					

*Note.* The “cases” in this analysis are the nine different types of SH/C experiences. For the last variable (6), only seven cases could be used (due to a glitch in survey construction, there was no data collected for the items “Receiving unsolicited pornographic material” and “Distributing intimate material [...]”). However, the data for each SH/C type and variable reflect information obtained from hundreds of participants.

A number of findings were noteworthy here (we will index the correlations in Table 8 by row and column, to make it easier to follow the discussion): First, the frequencies of actual SH/C offences reported in Paths A and B correlated almost perfectly with one another, *r* = .99 (Row: 1, Column: 3). This means that the relative frequencies with which different SH/C types were reported were basically the same for people who had been targets themselves and for people who said they only knew someone who had been a target. Given that this correlation was based on reports by two independent samples of participants, it yields very strong evidence for the validity of these reports – there seems to be a strong regularity at play in terms of which offences are committed more often than others.

Second, in Path C the expected reporting rates for types of offences experienced by oneself and offences experienced by others were almost perfectly correlated, too, *r* = .98 (Row: 5, Column: 6). Third, there were strong negative correlations (all *r* < -.92) between the reported frequencies of different types of offences in Paths A (Row 1) and B (Row 3), and the expected reporting rates in Path C (Columns 5 and 6). Thus, types of offences that actually were rarer were also judged—by participants with no knowledge of this data - as being more worthy of a formal report. Fourth, expected reporting (Path C) also predicted actual reporting (Paths A and B) quite strongly, with correlations ranging from *r* = .68 to *r* = .93 (Row 2, Columns 5 and 6; Row 4, Columns 5 and 6). Taken together, these findings suggest the existence of some implicit concept of offence severity that was strongly shared among participants in all three subsamples (Paths A, B and C) and also closely tied to offence rarity.

We decided to use the expected self-reporting rates in Path C (Variable 5 in Table 8) as our measure of offence severity, given that this data (as opposed to the actual reporting rates obtained from participants in Paths A and B) is probably the least contaminated with additional considerations such as fear of potential retribution attempts etc. In other words, the more people in Path C indicated they would formally report some kind of SH/C if it happened to them, the more severe this kind of SH/C is considered to be. Accordingly, participants who indicated that they themselves had become a target of sexual harassment or coercion (Path A) were assigned the severity level (from Path C) of the most severe type of transgression that they said they had experienced. This overall severity level was then used to predict reasons for non-reporting. 

Table 7 (columns “separated by severity”) shows how the reasons that the participants in Path A gave for not reporting their own SH/C experiences varied with the overall severity of the respective SH/C type. Eight participants with severity level 95.6 (i.e., distribution of intimate material as the most severe category) were excluded to achieve reasonably stable estimates at all severity levels. We fitted quadratic functions to the percentages in Table 7, using overall severity as the predictor. Note that due to the option of endorsing multiple forms of SH/C as well as multiple reasons for not reporting in our questionnaire we are not able to draw connections between individual reasons for non-reporting and specific types of SH/C. We can only consider common occurrences. Also, these analyses were not pre-registered and should thus be interpreted as exploratory in nature. Specifically, *p*-values should not be interpreted in terms of strict confirmatory hypothesis tests.

The following findings were most noteworthy to us: First, uncertainty as to whether one’s experiences were severe enough to be reported varied with overall severity in a strongly curvilinear fashion, y = 65.838 + 0.808x -0.008x^2^, *F*(2,5) = 10.301, *R*^2^ = .805, *p* = .017. The uncertainty was highest in the middle of the severity continuum, which may seem expectable given that these types of offences lay somewhere *in between* mild and severe. Note, however, that even at the highest severity level, a sizable majority of participants (61.5%) endorsed this reason for non-reporting (Table 7).

Second, endorsement of the expectation that reporting would not lead to any consequences *increased* in an almost perfectly linear fashion with overall severity, y = 44.506 + 0.248x + 0.001x^2^, *F*(2,5) = 7.002, *R*^2^ = .737, *p* = .036. This increase might seem unexpected at first, because one would usually think that reporting more severe experiences should *more* likely lead to consequences. Our interpretation is that this reason for non-reporting became more important with severity because participants only even started *thinking* about possible consequences when offences were relatively severe and thus reporting them became a realistic idea. Note that, even at the highest severity level, the vast majority of participants (84.6%) endorsed this reason for non-reporting (Table 7).

Third, overall severity very strongly predicted whether participants would abstain from reporting because doing so would have been too emotionally painful. With this increase, the quadratic component was particularly strong, y = 16.808 - 1.047x + 0.013x^2^, *F*(2,5) = 12.566, *R*^2^ = .834, *p* = .011: expected emotional distress was much higher towards the upper end of the severity continuum. At the highest severity level, 46.2% of participants endorsed this reason for non-reporting (Table 7). In our view, this effect is especially noteworthy because it suggests that more severe experiences will be systematically *less* likely to be reported.

### Consequences of In-/formal Complaints

Table 9 concerns the consequences of SH/C reporting. In the vast majority of informal reports, the participants said they knew that the respective report had led to no consequences whatsoever, or that they did not know whether there had been any consequences. When reporting formally, still 60% of participants in Path A and 50% in Path B stated the latter. Most participants reported that the consequences following their report were internal solutions. With this term including many different behaviours, unfortunately, we did not ask the participants to further specify if they were satisfied with the quality of those internal solutions. Notably, one participant in Path A and two participants in Path B said there had been legal consequences to the reported SH/C. Contrasting that with the overall numbers of relatively severe experiences reported by participants in both paths (see Table 5) this yields a sobering picture regarding the effectiveness of sanctioning systems for SH/C, as not even half of reports lead to consequences while the quality of these is not further specified. To be fair, most of this lack of effectiveness may also be related to the low volume of reports made by the victims.

**Table 9:** Consequences of In-/formal Complaints

Consequence	Path A		Path B	
	Informal Complaint (*n* = 166)	Formal Complaint (*n* = 15)	Informal Complaint (*n* = 211)	Formal Complaint (*n* = 42)
Yes, internal solutions or consequences (e.g., mediation, informal discussion/ reminder)	17/166	5/15	32/211	10/42
Yes, consequences under labour/ disciplinary law (e.g., warning, transfer, dismissal)	1/166	0/15	1/211	7/42
Yes, consequences under criminal law (e.g., charges, conviction)	1/166	1/15	0/211	4/42
No, there were no consequences	118/166	3/15	119/211	6/42
I am not aware of any/ I don't know	21/166	4/15	53/211	11/42
Not clear yet	8/166	2/15	6/211	4/42

### Relationship With the Offender

In Paths A and B, we asked participants about the type of relationship that had existed between the target and the respective offender at the time the SH/C took place. Table 10 displays the results. Due to an oversight on our side, the respective questions were asked somewhat differently in Paths A and B: whereas in Path A participants could check several response options at once, in Path B only one option could be checked. Consequently, the percentages for participants in Path B add up to 100, whereas those in Path A add up to 152.2. Despite this being the case, the percentages in the two paths correlated at *r*(7) = .90, *p* = .001 with one another, which we interpret as evidence for the validity of these responses.

**Table 10:** Type of Relationship Between Target and Offender at the Time of the Sexual Harassment/ Coercion

Type	Path A	Path B
Peers (e.g., fellow students or colleagues without disciplinary authority)	301 (50.3%)	274 (39.4%)
Direct supervisors/superordinate	81 (13.5%)	60 (8.6%)
Lecturers with assessment/grading authority	122 (20.4%)	124 (17.8%)
Indirect supervisors, higher level supervisors and higher-level managers	52 (8.7%)	24 (3.4%)
Other persons at a higher level of the hierarchy who are not direct supervisors	68 (11.4%)	44 (6.3%)
Clients or external service providers	26 (4.3%)	14 (2.0%)
Unknown persons	179 (29.9%)	62 (8.9%)
I do not know exactly	41 (6.9%)	71 (10.2%)
Other	40 (6.7%)	23 (3.3%)
Of (participants that answered)	598	696

*Note.* Multiple answers possible in Path A but not Path B.

In this analysis we were especially interested in power differentials. In both groups of participants, the offending person was most often described as a peer, that is, a person at the same level of the university’s internal hierarchy (Path A: 50.3%, Path B: 39.4%). So, the largest number of SH/C instances in our sample did *not* occur within relationships marked by formal power differentials.

However, sizable numbers of participants described the offender as someone who held more power than they did – this applies to the next four categories in Table 10. In Path B (where only one category could be picked), these percentages added up to 36.1, while in Path A 323 of the 598 (54,0%) answers identified the offender as someone of higher power (again, note that multiple choice was possible here). So, organisational power differentials *were* present in many cases – but we did not assess whether these power differentials did play a role in triggering or facilitating the offender’s abusive behaviour.

## Discussion

The present study investigated experiences of sexual harassment and coercion among people studying and working at a large university in Germany. We surveyed an unusually large sample and addressed important issues such as the frequency with which different types of harassment are experienced, predictors of such experiences, and the functioning of the complaint system that is supposed to handle this type of problem. All hypotheses that we had formed and pre-registered based on our analyses with the exploration sample were confirmed with the significantly larger hold-out sample. However, the study yielded many more important insights beyond those tests. We will now address our major findings in detail, including some possible directions for future research.

### Major Findings

Overall, about 40 percent of the participants in our study said they had some personal knowledge of one or more experiences of sexual harassment and/or coercion, whereas about 60 percent said they did not. Of the 40 percent who said they had such knowledge, about half said they themselves had been the target (Path A) whereas the other half said someone else had been the target (Path B). By far the strongest predictor of reporting to have been the target of sexual harassment or coercion (i.e., choosing Path A) was female gender, which is in line with all previous research that we are aware of (List & Feltes, 2015; Mense et al., 2022). The odds ratio for this effect was 7.45, 99% CI [5.05, 10.99] in the hold-out sample.

A comparison with previous research also yields similar percentages for some of the individual types of harassment and coercion. For example, in the Germany-wide study of female students by Feltes and colleagues (2012), 6.6% and 18.3% of respondents respectively stated that they had been confronted with obscene jokes or comments about their own bodies, while in the present study the corresponding figure for women in Path A was 22.4%. The number of female respondents who reported to have unsolicitedly received pornographic material was 2.7% in the present study, which is again close to the 1.1% reported in the Feltes et al.’s study (2012). Furthermore, the proportion of female respondents who reported having been subjected to sexual coercion using physical force was 1,4% in our study, which falls near the respective range (0.6 to 1.1%) reported in the Feltes et al.’s study (2012). Taken together, these results show a remarkable degree of convergence, which might be interpreted as a sign of good generalisability. However, the overall percentage of female participants stating that they had been a target of some type of SH/C was much higher in the Feltes et al.’s study (54.7%), as compared to ours (26.4%). This discrepancy might be rooted in the question format which was more small-scale regarding different forms of SH/C. Doing so, as mentioned before, can lead to higher prevalence within a survey (Ilies et al., 2003).

Overall, the actual prevalence of SH/C in the present study was also considerably smaller than the ones that the same participants had provided as estimates. Our data do not permit a definitive answer as to the origin of this discrepancy, but three possibilities come to mind: One is that the actual prevalence we found were atypically low due to the COVID-19 pandemic which — at the time our study was conducted—had forced most participants to work and study from home for the most part of the past two years. We consider it plausible that the relative social isolation thus imposed may have significantly lowered the actual prevalence of SH/C experiences. Note that the average *estimated* SH/C prevalence in our study (47.8% for women) was not that different from the respective actual prevalence reported by Feltes et al. (2012, whose study was conducted unaffected by quarantines). However, due to the relatively imprecise ways in which we queried our participants regarding a possible effect of the COVID-19 pandemic (see Limitations), we cannot draw any firm conclusions in this regard. Another factor that may at least partly account for the difference between estimated and actual prevalence we found might be the recent developments of addressing the topic of sexual harassment more in Media and society (e.g., the Me Too movement) which could in turn make people overestimate its frequency. An alternative, however, might also be that persons with a heightened interest in the topic (and thus, possibly, exaggerated expectations regarding prevalence) were over-represented in our sample.

In line with the hypotheses that we pre-registered based on our own exploratory analyses, we were able to clearly confirm that women are assumed to become targets of sexual harassment more often than men (47.8% vs. 16.7%; Table 2). This aligns well with what we know about actual prevalences from other studies, so the average participant did seem to have a relatively accurate representation of this existing imbalance. Also as predicted, participants who later said they had become a target of SH/C themselves provided significantly higher prevalence estimates. This is an important finding in itself, as it suggests that any survey on the topic should consider the respondents’ own relevant experiences as a covariate.

Using the data from Path C, it was possible to derive an almost perfectly reliable measure of the relative *severity* of SH/C types, based on the respective hypothetical reporting rates. The more severe the different types of sexual harassment and coercion were perceived to be, the more rarely they occurred in our sample. We are not aware of any previous investigations showing such a close link between the perceived severity and the actual prevalence of different types of SH/C. We consider this finding particularly noteworthy given that the data underlying the correlation came from independent subsamples.

Even though more severe offences were indeed more rare, sizable numbers of participants in our hold-out sample (*n* = 4,333) said they had been - or knew of someone who had been - the target of some of the most severe types of SH/C, such as being pressured to engage in sexual activity (Path A: *n* = 52, Path B: *n* = 51), being offered favours/benefits in exchange for sex (Path A: *n* = 62, Path B: *n* = 59), use of physical force to assert sexual interests (up to and including rape) (Path A: *n* = 35, Path B: *n* = 36), or distribution of intimate material in which target is identifiable (Path A: *n* = 19, Path B: *n* = 26).

Perhaps the most striking findings of our investigation concern the apparent non-functioning of complaint mechanisms: First, there was a vast discrepancy between the percentages of SH/C experiences that participants in Path C (who had no actual knowledge of such experiences) said they *would* report and the percentages that, according to participants in paths A and B (who did have such knowledge), actually *were* reported. Even of the most severe experiences, a sizable majority (> 85%) remained unreported (Table 5). The finding is especially striking given that participants in Path C had anticipated reporting rates close to 100 percent for the very same types of SH/C.

Given this, it was important to determine the possible reasons behind this absence of reporting (“Silence”). We found the following three findings to be most noteworthy in this regard: First, even with the most severe types of SH/C, most participants in Path A who had abstained from reporting their experiences (84.6%, Table 7) said they did so because reporting would not have led to any consequences anyway. Unfortunately, this perception seems to be fairly accurate (see below). We did not ask participants any further as to *why* they did not expect reporting to have any consequences. However, we speculate that this might be rooted in, for example, the conviction that the people working for the respective complaint office have too little investigative and/or sanctioning power to be of any help, and/or in the idea that the institution may be inclined to shove allegations “under the rug” in order to protect its reputation. Future studies should shine a more detailed light on these possibilities, and on ways of addressing these potential hindrances to a proper functioning of complaint systems.

Second, even with the most severe types of SH/C, most participants who had abstained from formally reporting their experiences (61.5%) said they had been uncertain as to whether their experiences were severe enough to be reported. We see several possible reasons for such doubts, which our data do not enable us to disentangle: These participants may have attributed part of the responsibility for what had happened to themselves (a very common reaction; Feltes et al., 2012) and/or they may have downplayed the event’s severity to themselves, in order to make the memory of what had happened more tolerable (a defence mechanism). A downplaying of severity may also be driven by a wish to make it feel less necessary to take action against the offender — because taking such action might lead to more uncontrollable and probably stressful consequences (McDonald, 2012). Again, future research should attempt to investigate these different possibilities in a more fine-grained manner. The possible perception that what one has experienced is viewed as more or less “normal” by many people and thus not worthy of being reported (i.e., an institutional culture that is relatively permissive regarding SH/C) should also be assessed as a potential impediment to reporting.

Third, as one might expect, there was a strong positive relationship between severity and a wish to avoid being reminded of the experience, which also seems to have made reporting less likely. Almost half of all participants with the most severe SH/C/ experiences (46.2%) endorsed this reason for not reporting, which is a sharp (quadratic) increase compared to most milder forms of harassment. Note that this effect makes it *less* likely for SH/C to be reported *the more severe they are* - in a way, offenders committing severe offences may thus “count on” their victims’ unwillingness to endure the significant emotional distress (e.g., shame) that would be associated with reporting. It seems that participants in Path C (i.e., those without any first- or second-hand knowledge of SH/C experiences) were unable to anticipate the impact of such factors, given that they predicted reporting rates close to 100% for the most severe offences.

In line with most participants’ expectations (see above), reporting one’s experiences as a target of SH/C often did lead to no real consequences. Unfortunately, this largely accurate perception may itself contribute to a kind of “downward spiral” in which potential complainants do not trust the existing complaint system and thus do not make use of it, which then renders the system even more ineffectual. What is worse, people’s reluctance to make use of an existing complaint system may be misinterpreted (e.g., by institutional leadership) as evidence that there are no problems worth complaining about - which then contributes to the persistence of the problems that *do* exist. However, given the fact that many of the complaints that *are* filed seem to go nowhere, one also cannot in good conscience recommend that potential complainants should just “have more trust” in the system. Clearly, the present study (and several others: e.g., Elson et al., 2021; Vazire & Holcombe, 2022) suggests that complaint mechanisms in academia may be in need of major reform.

### Limitations

The present study had a number of limitations. The most obvious of these is the lack of representativeness in regard to the overall population of students and employees at the surveyed university, or (German) universities more broadly. Although our sample size was unusually large, it is still possible that persons with a heightened interest in the topic field were over-represented in it, and this might have had an influence on the data we obtained. However, no study that we are aware of was ever able to survey a whole university, or many universities, in a representative fashion, so our study shares this limitation with all previous ones. We do think, however, that the relatively strong convergence between our findings and those of previous studies (e.g., regarding the relative prevalence of specific types of SH/C) may be interpreted as evidence in favour of validity.

Also, with regard to sample composition, we did not obtain any detailed information regarding the participants’ sexual orientation, disability, or other factors that were highlighted as additional risk factors in previous studies (e.g., Kammer-Kerwick et al., 2021; List & Feltes, 2015). The main reason we had to neglect these factors was data protection regulations at the university as well as the necessity to make the survey very brief.

For the second reason, our itemset omitted a few other important content domains that should be assessed in future studies: First, when asking about reasons for non-reporting, participants should be able to choose a response option stating that they themselves were able to confront the perpetrator, talk to them, and thus solve the problem. Several of our participants alluded to solutions of this kind in the free-response comment section that our survey contained. Likewise, the feeling that somehow oneself was partly responsible for bringing about one’s own SH/C experience (a common reaction in victims, as well as a popular defence by offenders) should be added as a response option in this area, and so should the concern that it would be difficult or impossible to prove the factual accuracy of one’s allegations (e.g., in court). Furthermore, we did not ask participants who had formally reported their experiences whether the offender *had* made an attempt to retaliate and, if so, how “successful” that attempt had been. This should be assessed in future studies, as well. When asking participants to *estimate* the prevalence of SH/C, it will be helpful to let them do so separately for different types of harassment and coercion. Also, our question regarding the possible influence of the COVID-19 pandemic was not phrased well, making it difficult to determine the influence of this extraneous factor on the data.

Finally, it will be necessary to shed more light on the *actual* role that power imbalances play in SH/C at academic institutions. In the introduction to this article, we highlighted the presence of a few risk factors that may be viewed as a breeding-ground for SH/C behaviour. For example, power differentials in academia are often steep - especially steep in Germany (Kreckel, 2016; Ohm, 2023) - and the most powerful positions are usually held by men (who constitute the vast majority of offenders; Statistisches Bundesamt, 2021; Mense et al., 2022). All of this, however, does not answer the question of how relevant these factors are in bringing about actual SH/C behaviour. An additional relevant factor might be the relative proximity and contact time between potential offenders and others, but the present study does not permit any conclusions regarding its influence. To overcome these limitations, future research should zoom in more specifically on this key aspect of the topic, and ask about the various *sources of power* that perpetrators have, and about the use they (do or do not) make of their power if they commit acts of SH/C. Of similar interest will be to elucidate the measures that perpetrators take to avoid detection, to silence victims, and to deflect allegations once they are made.

### Practical Recommendations

The following practical recommendations may be given: First, we need to take a harder look at whether and why the existing complaint systems do not seem to properly function the way they are supposed to. Their mere *existence* is simply not sufficient if it neither helps prevent many instances of SH/C nor leads to sanctions against most actual offenders. To understand this lack of functioning, it will be necessary to survey potential users in more detail regarding their perceptions of and experiences with the existing complaint systems. Research does suggest that complaint systems dedicated to other types of problematic behaviour in academia (e.g., scientific misconduct) suffer from similar ineffectiveness (Elson et al., 2021). If it turns out that self-corrective mechanisms within academic institutions have little chance of ever functioning well (e.g., due to the institutions’ interest to protect their reputation by minimising accusations or even “shoving them under the rug” completely), they will have to be ultimately replaced by ones that are more “external” (i.e., independent of the institution concerned).

A second recommendation follows directly from one of the unfortunate side-effects of the aforementioned failure of complaint systems to function properly. If the majority of potential complainants keep silent, and those who do not have little to no palpable success with their complaints, this may promote a false narrative in which “there is nothing to see here”. This of course applies to all sorts of misconduct, not just SH/C. In the case of SH/C, however, the present study suggests that the relevance of the issue may be particularly underestimated because of its sensitive nature and victims’ tendency to keep their experiences to themselves, to avoid further emotional distress. Perversely - but understandably - this avoidance may actually *increase* with the severity of one’s SH/C experiences. Therefore, in order to break these taboos, we suggest that academic institutions need to raise awareness of the SH/C issue more proactively. We believe that awareness training should become part of the routine onboarding process for all new employees and students at academic institutions, and maybe even be repeated on regular bases. Such training should cover (a) different types of SH/C, (b) relevant legal regulations, and (c) existing complaint mechanisms both within and outside of the institution. Based on the findings of the present study, we also recommend explicitly addressing the sensitive nature of the topic and the associated tendency of victims to keep silent about their experiences, which may even increase with how traumatising these experiences were. However, research into the effectiveness of such training is relatively scarce so far and has yielded mixed results (Roehling & Huang, 2018). Therefore, we believe that a continuous evaluation of the success of such programs is necessary. Furthermore, efforts to prevent or at least limit SH/C in academia may also benefit from implementation of measures that are not specifically tailored to target this particular issue, but broader ones such as conflict management, socio-emotional skills, gender equality and employee empowerment (McDonald et al., 2015).

## Supplementary Materials

The Supplementary Materials contain the following items (for access see Index of Supplementary Materials below):

- Preregistration Protocol
- Questionnaire (by M. Hoebel, A. Durglishvili, J. Reinold, D. Leising)
- Additional Results (Table 4a, Table 5a)

HoebelM.
DurglishviliA.
ReinoldJ.
LeisingD.
 (2022). Supplementary materials to "Sexual harassment and coercion in German academia: A large-scale survey study"
[Pre-registration protocol]. PsychOpen. https://osf.io/3uma5
10.5964/sotrap.9349PMC1178943339902154

HoebelM.
DurglishviliA.
ReinoldJ.
LeisingD.
 (2022). Supplementary materials to "Sexual harassment and coercion in German academia: A large-scale survey study"
[Questionnaire and additional results]. PsychOpen. 10.23668/psycharchives.12211
PMC1178943339902154

## Data Availability

The Data is unavailable due to the sensitive nature of the data and to uphold the consent statements that the data would not be shared with anyone but the research team. The complete data will be stored in a persistent fashion at a secure server located at TU Dresden.

## References

[sp1_r1] Hoebel M. Durglishvili A. Reinold J. Leising D. (2022). Supplementary materials to "Sexual harassment and coercion in German academia: A large-scale survey study" [Pre-registration protocol]. PsychOpen. https://osf.io/3uma5 10.5964/sotrap.9349 PMC11789433 39902154

[sp1_r2] Hoebel M. Durglishvili A. Reinold J. Leising D. (2022). Supplementary materials to "Sexual harassment and coercion in German academia: A large-scale survey study" [Questionnaire and additional results]. PsychOpen. 10.23668/psycharchives.12211 PMC11789433 39902154

